# Cross-sectional and prospective associations of sleep, sedentary and active behaviors with mental health in older people: a compositional data analysis from the Seniors-ENRICA-2 study

**DOI:** 10.1186/s12966-021-01194-9

**Published:** 2021-09-16

**Authors:** Verónica Cabanas-Sánchez, Irene Esteban-Cornejo, Esther García-Esquinas, Rosario Ortolá, Ignacio Ara, Irene Rodríguez-Gómez, Sebastien F. M. Chastin, Fernando Rodríguez-Artalejo, David Martínez-Gómez

**Affiliations:** 1 IMDEA Food Institute, CEI UAM+CSIC, Madrid, Spain; 2 PROFITH “PROmoting FITness and Health through physical activity” Research Group, Department of Physical and Sports Education, Faculty of Sport Sciences, University of Granada, Granada, Spain; 3 Department of Preventive Medicine and Public Health, Universidad Autónoma de Madrid and Idipaz, Madrid, Spain; 4 CIBER of Epidemiology and Public Health (CIBERESP), Madrid, Spain; 5 GENUD Toledo Research Group, Universidad de Castilla-La Mancha, Toledo, Spain; 6 Biomedical Research Networking Center on Frailty and Healthy Aging (CIBERFES), Madrid, Spain; 7 School of Health and Life Science, Institute for Applied Health Research, Glasgow Caledonian University, Glasgow, UK; 8 Department of Movement and Sport Sciences, Ghent Univeristy, Ghent, Belgium

**Keywords:** Depression, Loneliness, Happiness, Elderly, Compositional data analysis, Prospective

## Abstract

**Background:**

Most studies on the effects of sleep, sedentary behavior (SB), and physical activity (PA) on mental health did not account for the intrinsically compositional nature of the time spent in several behaviors. Thus, we examined the cross-sectional and prospective associations of device-measured compositional time in sleep, SB, light PA (LPA) and moderate-to-vigorous PA (MVPA) with depression symptoms, loneliness, happiness, and global mental health in older people (≥ 65 years).

**Methods:**

Data were taken from the Seniors-ENRICA-2 study, with assessments in 2015–2017 (wave 0) and 2018–2019 (wave 1). Time spent in sleep, SB, LPA and MVPA was assessed by wrist-worn accelerometers. Depression symptoms, loneliness, happiness, and global mental health were self-reported using validated questionnaires. Analyses were performed using a compositional data analysis (CoDA) paradigm and adjusted for potential confounders.

**Results:**

In cross-sectional analyses at wave 0 (*n* = 2489), time-use composition as a whole was associated with depression and happiness (all *p* < 0.01). The time spent in MVPA relative to other behaviors was beneficially associated with depression (γ = -0.397, *p* < 0.001), loneliness (γ = -0.124, *p* = 0.017) and happiness (γ = 0.243, *p* < 0.001). Hypothetically, replacing 30-min of Sleep, SB or LPA with MVPA was beneficially cross-sectionally related with depression (effect size [ES] ranged -0.326 to -0.246), loneliness (ES ranged -0.118 to -0.073), and happiness (ES ranged 0.152 to 0.172). In prospective analyses (*n* = 1679), MVPA relative to other behaviors at baseline, was associated with favorable changes in global mental health (γ = 0.892, *p* = 0.049). We observed a beneficial prospective effect on global mental health when 30-min of sleep (ES = 0.521), SB (ES = 0.479) or LPA (ES = 0.755) were theoretically replaced for MVPA.

**Conclusions:**

MVPA was cross-sectionally related with reduced depression symptoms and loneliness and elevated level of happiness, and prospectively related with enhanced global mental health. Compositional isotemporal analyses showed that hypothetically replacing sleep, SB or LPA with MVPA could result in modest but significantly improvements on mental health indicators. Our findings add evidence to the emerging body of research on 24-h time-use and health using CoDA and suggest an integrated role of daily behaviors on mental health in older people.

**Supplementary Information:**

The online version contains supplementary material available at 10.1186/s12966-021-01194-9.

## Background

The world population is ageing very swiftly [1]. Ageing is a complex process of physical, psychological and social changes that leads to physical decline and increase risk of mental health conditions [2]. The World Health Organization has estimated that approximately 15% of people aged 60 years and older suffer from a mental disease [1]. Concerns have focused on more severe disorders (e.g., depression) [3], but other relevant mental health conditions, such as loneliness and a low level of happiness, are highly prevalent in old age [4]. Identifying modifiable factors associated with these mental conditions in older adults is essential to promote healthy aging.

There is a large scientific literature about the benefits of physical activity (PA) on mental health in older adults [5]. Also, many studies have addressed the impact of sedentary behavior (SB) and sleep on mental health indicators in this age group [6–9]. Because time is finite during the day (i.e., 24-h), time spent in one behavior is inherently intertwined with time spent in the other activities [10]. Thus, it is important to assess the whole 24-h time-use, and utilize appropriate statistical methods accounting for the co-dependent nature of behaviors. Compositional Data Analysis (CoDA) allows the simultaneous examination of all time-use behaviors by creating a set of log ratios that contain relative information about all behaviors in the 24-h composition and enables accurate interpretation of the behaviors as proportions of total time-use [10, 11]. Unfortunately, most previous research failed to account for the compositional nature of time-use; in fact, we are aware of only a few studies, with a cross-sectional design, which used CoDA to investigate the association of time-use composition and mental health in older people [9, 12–15].

Moreover, there is only limited knowledge, proceeding from small number of individuals selected just before the retirement age or from small clinic-based samples, on how time-use composition are longitudinally associated with changes in mental health among older people [16, 17]. Therefore, using a CoDA approach, we aimed to: (i) examine the cross-sectional associations of accelerometer-derived sleep, SB, light physical activity (LPA) and moderate-to-vigorous physical activity (MVPA) with mental health indicators (i.e., depression symptoms, loneliness, happiness and global mental health) in older people; and (ii) to assess the prospective relationships between time-use composition and changes in mental health indicators in this same population.

## Methods

### Study design and participants

Data for this study were taken from the Seniors-ENRICA-2 cohort, formed by 3,273 community-dwelling individuals aged ≥ 65 years, enrolled between 2015 and 2017 (wave 0—baseline) in the city of Madrid and four large adjacent cities (Getafe, Torrejón, Alcorcón and Alcalá de Henares) [18, 19]. In these cities, participants were selected by sex- and district-stratified random sampling of all people holding a national healthcare card (data available at: http://www.comunidad.madrid/servicios/salud/tarjeta-sanitaria). Among those invited (*n* = 6418), 51% accepted to participate in the baseline assessments. Between 2018 and 2019 (wave 1), participants were invited to update the study information. During follow-up, 46 participants died and 1333 were lost, leading to a sample size of 1,894 individuals with wave 0 and wave 1 evaluations.

In the Seniors-ENRICA-2 study, data at both wave 0 and wave 1 were collected in three sequential stages: a) a computer-assisted telephone interview on health status and morbidity, lifestyle habits, and healthcare services use and knowledge; b) a first home visit for performing a complete physical exam, placing accelerometers on the wrist of participants, and collecting blood and urine samples; and, c) a one week-apart second home visit for completing an electronic dietary history and removing the accelerometers.

Study participants and their relatives provided informed written consent, and the study protocol was approved by the Clinical Research Ethics Committee of the *La Paz* University Hospital, in Madrid. All researchers involved in data collection received appropriate training and were certified before starting the fieldwork.

### Assessment of 24-h time-use composition

Time use composition was determined as the proportions of time spent on sleep, SB, LPA and MVPA objectively assessed by accelerometers. Participants wore an ActiGraph GT9X (ActiGraphInc, Pensacola, FL, USA), attached to the non-dominant wrist using a watch band, for seven consecutive days; they were asked to only remove it during water-based activities (i.e., shower, bath or swimming).

The complete protocol for accelerometer data analysis has been detailed elsewhere [18]. Briefly, raw accelerometer data at wave 0 were processed using the GGIR package (v. 1.7–0, https://cran.r-project.org/web/packages/GGIR/) implemented in R [20]. Sleep periods were detected utilizing an automatized algorithm [18, 21]. Awake time was classified into SB and PA intensities using a previously proposed threshold for ENMO (Euclidean Norm Minus One) in the non-dominant wrist: < 45 mg for SB, ≥ 45 mg and < 100 mg for LPA, and ≥ 100 mg for MVPA [22]. Days when the accelerometer registered at least 16 h were considered as valid, and only results from participants with at least 4 valid days (minimum one weekend day) were included in the analyses.

### Mental health outcomes

Depression was ascertained using the 10-item version of the Geriatric Depression Scale (GDS-10) [23]. The following yes/no questions were considered: (1) “Are you basically satisfied with your life?”; (2) “Have you dropped many of your activities and interests?”; (3) “Do you feel that your life is empty?”; (4) “Are you afraid that something bad is going to happen to you?”; (5) “Do you feel happy most of the time?”; (6) “Do you often feel helpless?”; (7) “Do you feel you have more problems with memory than most?”; (8) “Do you feel full of energy?”; (9) “Do you feel that your situation is hopeless?”; and (10) “Do you think that most people are better off than you are?”. The negative responses to questions 1, 5 and 8, and the number of positive responses to questions 2, 3, 4, 6, 7, 9 and 10 were summed to calculate a depressive symptoms score (range 0 to 10), with higher scores corresponding to higher levels of depression.

Loneliness was assessed with the three-item loneliness scale [24], which was developed specifically for telephone interviews in large surveys. This scale includes the following three questions: (1) How often do you feel that you lack companionship? (2) How often do you feel left out?, and (3) Hw often do you feel isolated from others?, with response options of *hardly ever* (coded as 1), *some of the time* (coded as 2) and *often* (coded as 3). The sum of three items provides a score ranging from 3 to 9, with higher scores indicating more loneliness.

Participants reported their level of happiness through the Cantril Ladder of Life Scale [25]. This instrument uses the image of a ladder with steps numbered from zero at the bottom to ten at the top, where the bottom represents the worst possible life and the top the best one. Participants were asked to answer the following question: “On which step of the ladder do you feel you personally stand at the present time?”. Thus, score ranged from 0 to 10 with higher values representing a higher happiness state.

Global mental health was assessed using the Spanish version of the 12-item Short Form Health Survey (SF-12, version 2) [26]. Participants rated their health, the extent to which their health limited daily activities and their emotional experience during the past 4 weeks, and the mental component summary (MCS) was calculated following the recommendations of the SF-12 developers [26], so that it was standardized to a norm with a mean of 50 and a standard deviation of 10 [27]. Thus, scores ranged from 0 to 100; higher scores in the MCS indicate better mental health, with a 2-point and an 8-point difference deemed to be, respectively, a small and a moderate-to-large difference [27].

These scales were applied at both wave 0 and wave 1. Changes in each mental health indicator (i.e., depression, loneliness, happiness, and global mental health) were calculated by subtracting the values at wave 0 from the values at wave 1 (e.g., change in depression was calculated as GDS-10 score at wave 1 minus GDS-10 score at wave 0).

### Potential confounders

Self-reported information was collected on sex, age, educational level (less than primary, primary, secondary, or university studies), marital status (single, married, separated, or widowed), household economy (difficult home economy: have great deal of difficulty, difficulty, or some difficulty making ends meet; or easy home economy: have some easy, easy, or great of easy making ends meet), tobacco smoking (current, former or never smoker), and alcohol consume (heavy, moderate, former or never). Food consumption was obtained through a validated computer-assisted face-to-face diet history, and total energy intake (kcal/day) was estimated with standard food composition tables [28]. Height and weight were measured by standardized procedures and body mass index (BMI) was calculated as weight (kg) divided by squared height (m^2^). Participants were also asked to report diseases diagnosed by a physician including diabetes mellitus, cardiovascular disease (ischemic heart disease, stroke or heart failure), hypertension, chronic respiratory disease (asthma or chronic bronchitis), osteomuscular disease (osteoarthritis, rheumatoid arthritis or hip fracture), neurodegenerative disease (Parkinson, or dementia/Alzheimer) and cancer at any site. The number of conditions was classified as none, one, and two or more diseases. Cognitive function was assessed with the Mini-Mental State Examination (MMSE) [29], which has been validated for use in Spanish geriatric population [30]. Finally, usual gait speed, a strong marker for physical function in older people, was evaluated by the 8-feet test, following the standardized protocol from the Short Physical Performance Battery [31]; gait speed was coded as: (0) unable; (1) ≤ 0.43 m/s; (2) 0.44–0.60 m/s; (3) 0.61–0.77 m/s; (4) ≥ 0.78 m/s.

### Statistical analyses

All analyses and graphical representations were performed with the statistical software R v.3.6.3 (http://cran.rproject.org), using *robCompositions* package. Statistical significance was set at *p* < 0.05. As recently recommended [32], Directed Acyclic Graph (DAG) was developed to completely reflect the relational and causal assumptions of the present study (supplementary Figure 1). DAG was created based on the compositional causal inference framework proposed by Arnold et al. [33] and recent relevant research on physical activity [3–5, 34, 35].

Data were analyzed following the principles of CoDA, proposed by Chastin et al. [10]. The compositional mean was computed calculating the geometric mean for each behavior, and then normalizing the data to the same constant (in this case, 100) and expressing the parts as proportions of the complete 24-h. The distribution of the time composition was illustrated by a matrix of ternary plots with three behaviors represented at a time. The dispersion of the compositional data set was estimated using the variation matrix that summarizes the variability structure of data by means of the log-ratio variance of all pair-wise behaviors. Values close to zero imply that two parts in the ratio are highly co-dependent. We did not need to address the issue of zeros in compositional data, because all participants spent some time in every time-use behavior.

In order to correctly treat and interpret the compositional daily-behaviors information, data contained in parts of the composition was firstly expressed relative to the other parts as log ratios [10]. Later, time spent in sleep, SB, LPA and MVPA was transformed into three isometric log ratio (*ilr*) coordinates, applying the structure of the following equations:

$${z}_{1}=\sqrt{\frac{3}{4}} \mathit{ln}\left(\frac{Sleep}{\sqrt[3]{SB*LPA*MVPA}}\right)$$

$${z}_{2}=\sqrt{\frac{2}{3}} \mathit{ln}\left(\frac{SB}{\sqrt[2]{LPA*MVPA}}\right)$$

$${z}_{3}=\sqrt{\frac{1}{2}} \mathit{ln}\left(\frac{LPA}{MVPA}\right)$$

For cross-sectional analyses, we fitted regression models using time-use composition (expressed as a set of *ilr* coordinates) as explanatory variables and the mental health indicators at wave 0 as response variables. Similarly, for prospective analyses, the set of *ilr* coordinates of the time-use composition was used as explanatory variables, while the change in mental health indicators was considered as dependent variables in regression models. Three models were created with progressive adjustment for potential confounders. Model 1 was adjusted for sex and age; model 2 was additionally adjusted for educational level, marital status, and household economy; and model 3 was further adjusted for smoking status, alcohol consume, total energy intake, BMI, cognitive function (MMSE score), physical function (gait speed test score), and the number of chronic diseases. In prospective analyses, regression models were additionally adjusting for baseline values of each mental health indicator, as appropriate.

Compositional isotemporal substitution analyses, using the above explained regression models, were applied to predict the expected change in outcomes when a fixed duration of time (i.e., 30 min) was reallocated from one behavior to another one, while the time in the remaining behaviors was kept constant [36]. The hypothetical change on each mental health indicator (effect size, ES) was estimated for reallocation of time between all possible pair-wise component combinations, in a cross-sectional and prospective approach.

Following the STROBE guidelines [37], a sensitivity analysis was conducted for each model by randomly removing 10% of the cases and checking for a significant shift in the results; no significant changes were observed. An additional sensitivity analysis was performed analyzing the cross-sectional associations including only participants from the prospective sample. Moreover, since participants with moderate or severe mental impairment might have difficulty self-reporting mental health information, a supplementary sensitivity analysis was executed examining the main cross-sectional and prospective associations after excluding participants with MMSE score < 24.

## Results

### Participants’ characteristics

From the 3,273 participants in the Seniors-ENRICA-2 study, 2,489 provided valid accelerometer data and complete information on potential confounders and, at least, one mental health scale at wave 0, forming the total analytic sample for this study (cross-sectional sample; Supplementary Figure 2). The sub-sample used for prospective analyses consisted of 1,679 participants with comprehensive data at wave 0 and wave 1 (prospective sample; Supplementary Figure 2). The average time between waves was 2.31 ± 0.31 years.

Table 1 shows the main characteristics of the study participants. Briefly, in the cross-sectional sample, the majority of participants were women (53.07%), married (66.05%), never smokers (52.51%), and moderate drinkers (69.79%); most had primary educational level (48.21%), easy home economy (85.74%), and one chronic disease (38.57%); the mean total energy intake, BMI and cognitive function score was 1944.99 kcal/day, 27.76 kg/m^2^ and 27.99 points, respectively; and most obtained the highest score on the gait speed test (69.55%). Slight differences between the cross-sectional and prospective sample were found for educational level, cognitive function, depression score, and relative compositional time spent in MVPA.

**Table 1:** Descriptive characteristics of the cross-sectional and prospective study sample at wave 0

	Cross-sectional sample	Prospective sample	*P*
n^a^	2489	1679	
Age (years)	71.68 ± 4.33	71.40 ± 4.15	*0.054*
Sex, n (%)			0.383
Men	1168 (46.93)	811 (48.30)	
Women	1321 (53.07)	868 (51.70)	
Educational level, n (%)			**0.015**
No studies	384 (15.43)	214 (12.75)	
Primary education	1200 (48.21)	792 (47.17)	
Secondary education	450 (18.08)	314 (18.70)	
University or higher education	455 (18.28)	359 (21.38)	
Marital status, n (%)			0.891
Single	164 (6.59)	114 (6.79)	
Married	1644 (66.05)	1117 (66.53)	
Separated	173 (6.95)	121 (7.21)	
Widowed	508 (20.41)	327 (19.48)	
Household economy, n (%)			*0.093*
Difficult economy	355 (14.26)	209 (12.45)	
Easy economy	2134 (85.74)	1470 (87.55)	
Smoking status, n (%)			0.766
Current	233 (9.36)	154 (9.17)	
Former	949 (38.13)	659 (39.25)	
Never	1307 (52.51)	866 (51.58)	
Alcohol consume, n (%)			0.706
Heavy drinker^b^	116 (4.66)	86 (5.12)	
Moderate drinker	1737 (69.79)	1188 (70.76)	
Former	167 (6.71)	106 (6.31)	
Never	469 (18.84)	299 (17.81)	
Total energy intake (kcal/day)	1944.99 ± 345.03	1955.57 ± 344.49	0.331
Body Mass Index (kg/m^2^)	27.76 ± 4.42	27.70 ± 4.36	0.691
Chronic diseases, n (%)			0.308
No conditions	489 (19.65)	359 (21.38)	
One condition	960 (38.57)	650 (38.71)	
Two or more conditions	1040 (41.78)	670 (39.90)	
Cognitive function (MMSE)	27.99 ± 2.00	28.20 ± 1.68	** < 0.001**
Physical function – gait speed test score, n (%)			0.412
Score 0 (unable)	7 (0.28)	2 (0.12)	
Score 1 (≤ 0.43 m/s)	90 (3.62)	47 (2.80)	
Score 2 (0.44–0.60 m/s)	224 (9.00)	144 (8.58)	
Score 3 (0.61–0.77 m/s)	437 (17.56)	310 (18.46)	
Score 4 (≥ 0.78 m/s)	1731 (69.55)	1176 (70.04)	
Depression score (GDS-10)	0.93 ± 1.76	0.80 ± 1.58^d^	**0.012**
Loneliness score (3-items loneliness scale)	3.69 ± 1.35	3.68 ± 1.35^d^	0.843
Happiness score (0–10)	7.49 ± 1.75	7.56 ± 1.69^d^	0.196
Global mental health (SF-12)	50.39 ± 9.68	50.94 ± 9.06^d^	0.067
Compositional time-use, geometric mean^c^ (%, min/day)			
Sleep	32.68 (470.59)	32.30 (465.12)	0.285
SB	54.07 (778.61)	53.86 (775.58)	0.697
LPA	9.95 (143.28)	10.24 (147.46)	*0.076*
MVPA	3.30 (47.52)	3.60 (51.84)	**0.018**

Values are mean ± SD, unless otherwise indicated

*P* = differences between cross-sectional and prospective sample, estimated by T-test for continuous variables and Chi-square test for categorical variables

Significant (*p* < 0.05) and borderline (*p* < 0.1) differences are in bold and italics, respectively

*Abbreviations*: *GDS* Geriatric Depression Scale, *LPA* Light Physical Activity, *MMSE* Mini-Mental State Examination, *MVPA* Moderate-to-Vigorous Physical Activity, *SB* Sedentary Behavior, *SD* Standard Deviation, *SF* Short Form

^a^n slightly varies in analyses for each mental health indicator

^b^Heavy drinker: ≥ 10 g/day in woman; ≥ 20 g/day in men

^c^Compositional geometric mean centered to 100. Values were also expressed as min/day (closed to a constant of 1440 min / day). Mean of total time registered by accelerometers was 1430.17 ± 6.52 min in the cross-sectional sample, and 1430.30 ± 6.26 min/day in the prospective sample

^d^Values (mean ± SD) for mental health indicators at wave 1 for prospective sample was: Depression score: 0.86 ± 1.57 (*p* = 0.061); Loneliness score: 3.76 ± 1.40 (*p* = 0.006); Happiness score: 7.42 ± 1.63 (*p* < 0.001); Global mental health: 50.35 ± 9.69 (*p* = 0.016). Differences in mental health indicators between wave 0 and wave 1 in prospective sample was estimated by paired T-test

**Fig. 1:**
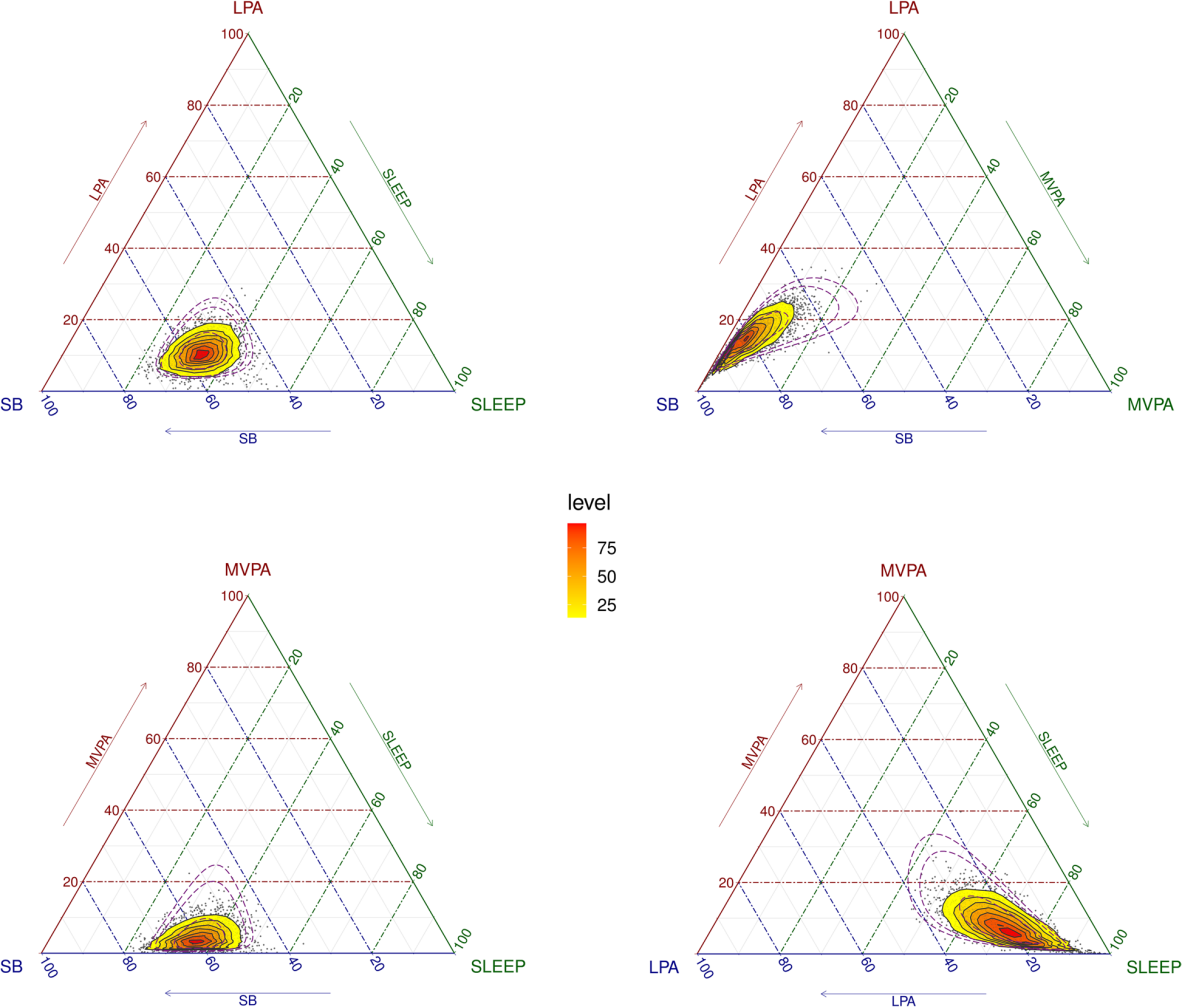
Ternary plots of the cross-sectional sample compositions of time spent in sleep, sedentary behavior (SB), light physical activity (LPA) and moderate-to-vigorous physical activity (MVPA), at wave 0. Heat map depicts the frequency distribution of compositions

Ternary diagrams plotting the compositional time-use distribution at wave 0 for the cross-sectional sample are presented in Fig. 1. Since a four-part composition is examined, the representation is performed in four diagrams containing a 3-dimensional representation of each 3-behavior combination. The vertices of the diagrams represent each behavior of the composition, so that points lying close to a vertex have high percentages of the behavior that is represented by that vertex, while points lying in the center of the triangle have equal percentages of all three behaviors [10]. Likewise, Supplementary Figure 3 illustrate time-use composition for the prospective sample at wave 0. According to geometric mean of the composition, participants in the cross-sectional sample spent 32.68% of daily time in sleep (470.59 min/day), 54.07% in SB (778.61 min/day), 9.95% in LPA (143.28 min/day), and 3.30% in MVPA (47.52 min/day; Table 1). The variability of the data is summarized by the variation matrix in Supplementary Table 1. The pair-wise log-ratio variances reflected the highest proportional co-dependence between sleep and SB, while time in MVPA was the least co-dependent on the other behaviors.

### Cross-sectional associations of time-use composition with mental health indicators

In the fully adjusted models for cross-sectional analyses (Table 2), time-use composition as a whole was significantly associated with depression (*p* < 0.001), and happiness (*p* < 0.001), whereas the relation with global mental health remained borderline (*p* = 0.075). The time spent in MVPA relative to other behaviors was beneficially associated with depression (γ = -0.397, *p* < 0.001), loneliness (γ = -0.124, *p* = 0.017) and happiness (γ = 0.243, *p* < 0.001). Results were similar when cross-sectional associations were analyzed including only the participants from the prospective sample (sensitive analysis; Supplementary Table 2).

**Table 2:** Cross-sectional associations between behaviors composition (proportion of the day spent in sleep, SB, LPA and MVPA) and mental health outcomes at wave 0 (cross-sectional sample)

	Composition model	Sleep		SB		LPA		MVPA	
	*p*	**γ**	*p*	**γ**	*p*	**γ**	*p*	**γ**	*p*
Depression (*n* = 2481)									
Model 1	** < 0.001**	0.342	*0.056*	0.083	0.642	0.136	0.308	-0.562	** < 0.001**
Model 2	** < 0.001**	0.278	0.116	0.086	0.623	0.151	0.250	-0.515	** < 0.001**
Model 3	** < 0.001**	0.250	0.151	-0.017	0.923	0.164	0.203	-0.397	** < 0.001**
Loneliness (*n* = 2489)									
Model 1	**0.007**	0.056	0.696	-0.008	0.958	0.113	0.279	-0.162	**0.003**
Model 2	**0.020**	0.007	0.957	-0.026	0.847	0.176	*0.077*	-0.158	**0.003**
Model 3	0.115	-0.021	0.873	-0.013	0.923	0.158	0.110	-0.124	**0.017**
Happiness (*n* = 2452)									
Model 1	** < 0.001**	-0.097	0.603	-0.229	0.219	-0.008	0.951	0.335	** < 0.001**
Model 2	** < 0.001**	-0.061	0.738	-0.209	0.253	-0.027	0.844	0.297	** < 0.001**
Model 3	** < 0.001**	-0.031	0.864	-0.174	0.339	-0.037	0.783	0.243	** < 0.001**
Mental health (*n* = 2443)									
Model 1	** < 0.001**	-1.418	0.172	0.235	0.817	-0.027	0.972	1.209	**0.002**
Model 2	**0.002**	-0.961	0.348	-0.111	0.912	0.131	0.860	0.941	**0.016**
Model 3	*0.075*	-0.754	0.458	-0.084	0.933	0.266	0.719	0.571	0.142

Values are shown as non-standardized gamma coefficients (γ) from CODA regression models; γ can be interpreted as the strength and direction of the association between the amount of time spent in one behavior relative to the others and the mental health outcome

Model 1 adjusted by sex, and age; model 2 adjusted as model 1 plus educational level (no studies, primary, secondary, university), marital status (single, married, separated, widowed), and household economy (difficult economy, easy economy); model 3 adjusted as model 2 plus smoking status (current, former, never), alcohol consume (heavy, moderate, former, never), total energy intake (kcal/day), BMI (kg/m^2^), cognitive function (mini-mental state examination score), physical function (gait speed test score: unable, ≤ 0.43 m/s, 0.44–0.60 m/s, 0.61–0.77 m/s, ≥ 0.78 m/s), and chronic diseases (0, 1, 2 +)

Statistically significant values are shown in bold (*p* < 0.05) and borderline significant values are shown in italics (*p* < 0.1)

*Abbreviations*: *SB* Sedentary Behavior, *LPA* Light Physical Activity, *MVPA* Moderate-to-Vigorous Physical Activity

Table 3 shows the theorical change expected on values of mental health at wave 0, when 30-min of time is reallocated between compositional time-use behaviors (fully adjusted models; results for models 1 and 2 are included in Supplementary Table 3). Mainly, replacing 30-min of Sleep, SB or LPA with MVPA was beneficially related with mental health indicators, with effect sizes ranging -0.326 to -0.246 for depression, -0.118 to -0.073 for loneliness, and 0.152 to 0.172 for happiness.

**Table 3:** Compositional isotemporal substitution analyses showing the hypothetical change on mental health indicators at wave 0 when 30-min of time is re-allocated between compositional behaviors

	Depression (*n* = 2481)	Loneliness (*n* = 2489)	Happiness (*n* = 2452)	Mental health (*n* = 2443)
	ES (95% CI)	ES (95% CI)	ES (95% CI)	ES (95% CI)
↑ Sleep				
↓ SB	0.013 (-0.018, 0.043)	0.000 (-0.023, 0.023)	0.006 (-0.025, 0.038)	-0.031 (-0.205, 0.143)
↓ LPA	**-0.067 (-0.130, -0.004)**	-0.045 (-0.093, 0.004)	0.020 (-0.046, 0.086)	-0.060 (-0.421, 0.300)
↓ MVPA	**0.507 (0.372, 0.642)**	**0.151 (0.044, 0.257)**	**-0.307 (-0.452, -0.163)**	-0.730 (-1.505, 0.045)
↑ SB				
↓ Sleep	-0.014 (-0.045, 0.018)	0.000 (-0.024, 0.024)	-0.006 (-0.038, 0.027)	0.034 (-0.145, 0.212)
↓ LPA	**-0.080 (-0.143, -0.017)**	-0.045 (-0.093, 0.004)	0.014 (-0.052, 0.080)	-0.029 (-0.389, 0.331)
↓ MVPA	**0.494 (0.364, 0.624)**	**0.150 (0.047, 0.253)**	**-0.313 (-0.453, -0.174)**	-0.699 (-1.445, 0.047)
↑ LPA				
↓ Sleep	0.051 (-0.002, 0.105)	0.036 (-0.004, 0.077)	-0.016 (-0.071, 0.040)	0.059 (-0.246, 0.363)
↓ SB	**0.065 (0.012, 0.117)**	0.036 (-0.004, 0.077)	-0.010 (-0.065, 0.045)	0.025 (-0.274, 0.325)
↓ MVPA	0.559 (0.388, 0.730)	**0.187 (0.052, 0.321)**	**-0.323 (-0.506, -0.141)**	-0.674 (-1.655, 0.307)
↑ MVPA				
↓ Sleep	**-0.260 (-0.330, -0.189)**	**-0.074 (-0.128, -0.019)**	**0.152 (0.078, 0.226)**	0.386 (-0.019, 0.790)
↓ SB	**-0.246 (-0.310, -0.182)**	**-0.073 (-0.123, -0.023)**	**0.158 (0.090, 0.226)**	0.352 (-0.016, 0.721)
↓ LPA	**-0.326 (-0.443, -0.209)**	**-0.118 (-0.209, -0.028)**	**0.172 (0.049, 0.295)**	0.323 (-0.345, 0.990)

Values are estimated modification on mental health indicators at wave 0 (effect size and 95% Confidence Interval) when 30 min were theoretically re-allocated from one behavior to another one, taking constant the time in the other behaviors

Analyses were adjusted by sex (male, female), age (years), educational level (no studies, primary, secondary, university), marital status (single, married, separated, widowed), household economy (difficult economy, easy economy), smoking status (current, former, never), alcohol consume (heavy, moderate, former, never), total energy intake (kcal/day), BMI (kg/m^2^), cognitive function (mini-mental state examination score), physical function (gait speed test score: unable, ≤ 0.43 m/s, 0.44–0.60 m/s, 0.61–0.77 m/s, ≥ 0.78 m/s), chronic diseases (0, 1, 2 +), and exposure outcome at baseline

Statistically significant values are shown in bold (*p* < 0.05)

*Abbreviations*: *ES* Effect Size, *SB* Sedentary Behavior, *LPA* Light Physical Activity, *MVPA* Moderate-to-Vigorous Physical Activity

### Prospective associations of time-use composition at wave 0 with change in mental health indicators

During the follow-up, depression (*p* = 0.061) and loneliness feelings (*p* = 0.006) increased, while the level of happiness (*p* < 0.001) and global mental health (*p* = 0.016; Table 1) decreased. The CoDA models analyzing the association of time-use composition at baseline with changes in mental health indicators over 2.31 years are reported in Table 4. For the fully adjusted models, time-use distribution as a whole was not significantly associated with the change in mental health indicators (all p ≥ 0.198). However, the time spent in MVPA relative to other behaviors was associated with favorable changes in global mental health (γ = 0.892, *p* = 0.049), while sleep time relative to the rest of behaviors was related to adverse changes in depression (γ = 0.347, *p* = 0.049).

**Table 4:** Prospective associations between behaviors composition (proportion of the day spent in sleep, SB, LPA and MVPA) at wave 0 and mental health indicators at wave 1 (prospective sample)

	Composition model	Sleep		SB		LPA		MVPA	
	*p*	**γ**	*p*	**γ**	*p*	**γ**	*p*	**γ**	*p*
Depression (*n* = 1675)									
Model 1	0.160	0.305	*0.088*	-0.274	0.115	0.080	0.532	-0.111	0.105
Model 2	0.220	0.280	0.115	-0.231	0.180	0.049	0.697	-0.099	0.147
Model 3	0.198	0.347	**0.049**	-0.326	*0.057*	0.049	0.695	-0.071	0.301
Loneliness (*n* = 1677)									
Model 1	0.467	0.035	0.819	-0.061	0.679	0.121	0.273	-0.094	0.111
Model 2	0.529	0.010	0.946	-0.040	0.789	0.117	0.286	-0.087	0.137
Model 3	0.518	0.008	0.956	-0.036	0.808	0.116	0.289	-0.088	0.133
Happiness (*n* = 1649)									
Model 1	0.501	0.051	0.805	-0.081	0.686	-0.073	0.625	0.103	0.197
Model 2	0.681	0.026	0.899	-0.023	0.907	-0.094	0.523	0.092	0.247
Model 3	0.637	-0.021	0.917	0.005	0.981	-0.079	0.589	0.096	0.224
Mental health (*n* = 1661)									
Model 1	0.140	-0.587	0.625	0.500	0.668	-0.947	0.271	1.034	**0.025**
Model 2	0.147	-0.536	0.653	0.354	0.760	-0.813	0.342	0.995	**0.030**
Model 3	0.254	-0.422	0.722	0.404	0.726	-0.874	0.304	0.892	**0.049**

Values are shown as non-standardized gamma coefficients (γ) from CODA regression models; γ can be interpreted as the strength and direction of the association between the time spent in one behavior relative to the others and the change in mental health outcome

Model 1 adjusted by sex (male, female), age (years), and exposure outcome at baseline; model 2 adjusted as model 1 plus educational level (no studies, primary, secondary, university), marital status (single, married, separated, widowed), and household economy (difficult economy, easy economy); model 3 adjusted as model 2 plus smoking status (current, former, never), alcohol consume (heavy, moderate, former, never), total energy intake (kcal/day), BMI (kg/m^2^), cognitive function (mini-mental state examination score), physical function (gait speed test score: unable, ≤ 0.43 m/s, 0.44–0.60 m/s, 0.61–0.77 m/s, ≥ 0.78 m/s), and chronic diseases (0, 1, 2 +)

Statistically significant values are shown in bold (*p* < 0.05) and borderline significant values are shown in italics (*p* < 0.1)

*Abbreviations*: *SB* Sedentary Behavior, *LPA* Light Physical Activity, *MVPA* Moderate-to-Vigorous Physical Activity

The hypothetical effects of reallocating 30-min between compositional time-use behaviors on the prospective change in mental health indicators are presented in Table 5 (fully adjusted models; results for model 1 and 2 are showed in Supplementary Table 4). We observed a beneficial prospective effect on global mental health when 30-min of sleep (ES = 0.521), SB (ES = 0.479) or LPA (ES = 0.755) were theoretically replaced for MVPA. Substituting SB for sleep was detrimentally related with change on depression (ES = 0.033).

**Table 5:** Compositional isotemporal substitution analyses projecting the theorical effect of reallocating 30-min of time between compositional behaviors on mental health indicators at wave 1

	Depression (*n* = 1675)	Loneliness (*n* = 1677)	Happiness (*n* = 1649)	Mental health (*n* = 1661)
	ES (95% CI)	ES (95% CI)	ES (95% CI)	ES (95% CI)
↑ Sleep				
↓ SB	**0.033 (0.002, 0.063)**	0.002 (-0.024, 0.029)	-0.001 (-0.037, 0.034)	-0.040 (-0.246, 0.166)
↓ LPA	0.001 (-0.060, 0.063)	-0.032 (-0.085, 0.021)	0.023 (-0.048, 0.094)	0.236 (-0.176, 0.648)
↓ MVPA	0.093 (-0.030, 0.215)	0.093 (-0.012, 0.198)	-0.101 (-0.241, 0.039)	**-0.962 (-1.783, -0.141)**
↑ SB				
↓ Sleep	**-0.033 (-0.065, -0.002)**	-0.002 (-0.029, 0.025)	0.001 (-0.035, 0.038)	0.041 (-0.170, 0.252)
↓ LPA	-0.031 (-0.091, 0.030)	-0.034 (-0.086, 0.019)	0.024 (-0.046, 0.095)	0.276 (-0.133, 0.683)
↓ MVPA	0.061 (-0.057, 0.178)	0.091 (-0.010, 0.191)	-0.100 (-0.234, 0.035)	**-0.923 (-1.71, -0.136)**
↑ LPA				
↓ Sleep	-0.006 (-0.058, 0.046)	0.026 (-0.019, 0.071)	-0.018 (-0.079, 0.042)	-0.186 (-0.537, 0.164)
↓ SB	0.028 (-0.023, 0.079)	0.028 (-0.016, 0.072)	-0.020 (-0.079, 0.039)	-0.228 (-0.569, 0.114)
↓ MVPA	0.088 (-0.068, 0.244)	0.119 (-0.015, 0.253)	-0.119 (-0.299, 0.060)	**-1.15 (-2.197, -0.103)**
↑ MVPA				
↓ Sleep	-0.060 (-0.127, 0.008)	-0.050 (-0.108, 0.009)	0.054 (-0.024, 0.132)	**0.521 (0.066, 0.975)**
↓ SB	-0.026 (-0.087, 0.036)	-0.047 (-0.100, 0.006)	0.053 (-0.018, 0.124)	**0.479 (0.066, 0.892)**
↓ LPA	-0.057 (-0.168, 0.054)	-0.081 (-0.176, 0.014)	0.077 (-0.051, 0.205)	**0.755 (0.011, 1.500)**

Values are estimated modification on mental health indicators at wave 1 (effect size and 95% Confidence Interval) when 30 min were theoretically re-allocated from one behavior to another one, taking constant the time in the other behaviors

Analyses were adjusted by sex (male, female), age (years), educational level (no studies, primary, secondary, university), marital status (single, married, separated, widowed), household economy (difficult economy, easy economy), smoking status (current, former, never), alcohol consume (heavy, moderate, former, never), total energy intake (kcal/day), BMI (kg/m^2^), cognitive function (mini-mental state examination score), physical function (gait speed test score: unable, ≤ 0.43 m/s, 0.44–0.60 m/s, 0.61–0.77 m/s, ≥ 0.78 m/s), chronic diseases (0, 1, 2 +), and exposure outcome at baseline

Statistically significant values are shown in bold (*p* < 0.05)

*Abbreviations*: *ES* Effect Size, *SB* Sedentary Behavior, *LPA* Light Physical Activity, *MVPA* Moderate-to-Vigorous Physical Activity

## Discussion

In this cohort of older people, the proportion of time spent in MVPA relative to other behaviors showed the largest associations with better mental health; in particular, MVPA was cross-sectionally related with reduced depression symptoms and loneliness and elevated level of happiness, and prospectively related with enhanced global mental health. Further, compositional isotemporal substitution analyses reveled that theoretically replacing sleep, SB or LPA with MVPA could result in improvements on mental health indicators. Our findings add evidence to the emerging body of research on 24-h time-use and health using CoDA, and suggest an integrated role of daily behaviors on mental health in older people; thus, promoting MVPA, to the detriment of other daily behaviors, could be an effective strategy for encouraging mental health in old age.

Application of CoDA has produced new evidence on the impact of the 24-h behaviors on a wide range of health outcomes [10, 38, 39]. However, only a few previous studies addressed the cross-sectional relationship between time-use and mental health, utilizing compositional methods [9, 12–15]. In a research with 430 middle-age participants (41.3 ± 11.7 years), accelerometer-derived activity composition was associated with physical health-related quality of life (QoL) but not with mental health-related QoL or symptoms of depression, anxiety or stress [12]. Further, two recent studies explored the cross-sectional relationship between 24-h movement behaviors (self-reported sleep duration, and accelerometer-based SB, LPA and MVPA) and mental health among 1095 Japanese workers (50.2 ± 9.5 years) [14] and 370 Swedish office workers (41 ± 9 years) [15], using CoDA procedures; in both, the relative time engaged in MVPA was not significantly associated with mental health outcomes. However, in our study, MVPA relative to the other behaviors showed a beneficial cross-sectional association with depression symptoms, loneliness feelings and happiness score. Notably, the previous studies included middle-age population, while ours focused on older people. It is plausible that the cross-sectional relationship of daily activity composition and mental health was stronger among the elderly, because in the Canadian Health Measure Survey, an association between higher proportions of device-measured MVPA and better mental health was found among those aged ≥ 65 years but not 18–64 years [9].

Significant results emerged in prospective analyses were limited, but we found MVPA, relative to the other behaviors, positively associated with changes in global mental health. The comparison with former reports is complicated because only two previous studies using CoDA have investigated the prospective association between daily behaviors and changes in mental health among older people, and showed discordant results. Lewthwaite et al. [16] found that the change in the 24-h behavior composition was associated with change in anxiety, but not in depression. However, the study sample only included 95 patients (70.5 ± 6.8 years) with chronic obstructive pulmonary disease, which limits the generalizability of results to the general older population. Conversely, Olds et al. [17] reported that changes in the time-use composition following retirement were related to conditional changes in depression, stress and self-esteem, but not to anxiety, well-being or life satisfaction, in 105 participants from the LAW (Life After Work) cohort. These two studies included small sample sizes and the use of time was ascertained by a 24-h recall tool (Multimedia Activity Recall for Children and Adults); thereby, ours is the first study using compositional methods to examine how accelerometer-derived daily behaviors prospectively associates with mental health in a relatively large cohort of older people.

Unexpectedly, the proportion of time in SB and LPA showed null relationships with mental health indicators in both cross-sectional and prospective analyses. LPA is the larger contributor to energy expenditure in the older population [40] and previous research based on traditional statistical analyses found stronger associations with mental health for LPA than MVPA [7, 8]. In contrast, former studies using CoDA found MVPA but not LPA to be associated with better mental [9] and cognitive [41] health in the elderly. It is plausible that the effect of LPA depends on the time accumulated in MVPA, so that sufficient levels of MVPA may attenuate associations of LPA with mental health. On the other hand, the literature has showed inconsistent relationships between SB and mental health. For example, some studies emphasized that high levels of device-measured SB increased the risk of depression in older people [42], while others found no association [43]. It has been suggested that different types of SB have distinct impacts on mental health, so that mentally passive SB (e.g., watching TV) shows detrimental associations, whereas mentally active SB (e.g., reading or internet use) seems to have beneficial or null associations with mental health in adults [44] and older adults [45, 46]. Finally, we only found a borderline detrimental association between sleep time, relative to other behaviors, with depression symptoms in prospective analyses. To date, the relationship between sleep and depression remains unclear. Several studies indicate that short sleep duration has a higher risk of development and recurrence of depression [47, 48]; however, a meta-analysis by Zhai et al. found a higher risk of depression for long sleep duration [6]. Future research should investigate the possible curvilinear and/or bidirectional association of sleep duration with mental health in older people.

At last, the compositional isotemporal substitution analyses performed in the present study suggested that replacing 30 min of sleep, SB, or LPA with MVPA is theoretically associated with modest but significant improvements in mental health of older people. The small effect sizes detected are consistent with previous estimates using compositional analyses. For example, in a cross-sectional study using a representative sample of 3233 US adults, Pozo et al. reported that reallocating 60 min from SB to MVPA was associated with significant but small (i.e., -0.09 points) theoretical reductions in depression symptoms [13]. In a longitudinal way, Lewthwaite et al. observed slight favorable changes in anxiety when 30 min was re-allocated to MVPA replacing SB (standardized ES = 0.070) or LPA (standardized ES = 0.056) [16]. It is important to highlight that the cohort used in our study is formed by non-clinical and non-institutionalized population and, therefore, a relatively healthy older population was evaluated. Recent research has advised that the impact of physical activity on mental health indicators may be stronger for clinical populations compared to non-clinical populations [49], which could partly explain the moderate effect sizes reported in the current study. Anyway, given the global burden associated with mental health decline in older people, our results -although showing modest effect sizes-, could be clinically relevant, and suggest that addressing the distribution of daily behaviors (i.e., increasing MVPA) could be an effective approach to promote mental health in older people.

### Strengths and limitations

The major strength of this study was the application of CoDA, which enabled accounting for the codependence between time-used in behaviors, as well as the objective assessment of daily behaviors across 24-h with accelerometry. Further strengths were the prospective design of the study, the relatively large sample, and the analytical adjustment for a high number of potential confounders. Moreover, linking the compositional time-use at baseline to changes in mental health over time (adjusting for baseline values of mental health indicators) reinforces the advantages of the prospective design, intended to establish the temporality of the associations and reducing reverse causation. The limitations of the study include the relatively short follow-up period, and the observational nature of the study, which does not allow for entirely discarding residual confounding. Further, we used validated tests to assess a wide range of mental health constructs, but the diagnoses by a physician were not available. Nevertheless, to address the possible difficulty of participants with mental impairment in self-report mental health information and to test whether this might affect the results, we rerun the main analyses after excluding participants with an MMSE score < 24, but we obtained similar estimates (Supplementary Table 5). Finally, our results do not permit to establish the optimal doses of each time-use behavior to maintain or improve mental health in older people.

## Conclusion

The results of the present study showed that time-use composition was related to mental health in older people. Mainly, time spent in MVPA relative to other behaviors was beneficially associated with depression, loneliness, and happiness in cross-sectional analyses, and with global mental health in prospective analyses. Moreover, compositional isotemporal analyses showed that hypothetically replacing sleep, SB or LPA with MVPA could result in modest but significantly improvements on mental health indicators. Therefore, interventions and recommendations aimed at increasing MVPA in older people could provide beneficial effects on mental health. However, future experimental research should address the identification of effective and realistic strategies to modify daily behavior components from a holistic approach, in order to promote healthy mental aging.

## Supplementary Information

**Additional file 1: Supplementary Figure 1.** Directed Acyclic Graph (DAG) illustrating the relational and causal assumptions of the present study. *Diagnosed health conditions included in our model were: cardiovascular disease, hypertension, diabetes mellitus, chronic respiratory disease, osteomuscular disease, neurodegenerative disease, and cancer at any site. The covariates node was created to show all confounding variables including in the model that have a potential relationship with both the 24-h composition and the present and future mental health indicators. Some relationships between factors were omitted for clarity.

**Additional file 2: Supplementary Figure 2.** Flowchart of the analytical cross-sectional and prospective sample for the present study, from the Seniors-ENRICA-2 participants. ^†^The inclusion criteria were defined as having at least 4 valid days (at least, 1 valid weekend day; a valid day was considered as having at least 16 hours of record). *No significant differences were found between initial sample of Seniors-ENRICA-2 study and cross-sectional sample included in the present study in sex (46.87% vs. 46.93% of men, *p* = 0.965) or age (71.85±4.49 vs. 71.69±4.33, *p* = 0.161).

**Additional file 3: Supplementary Figure 3.** Ternary plots of the prospective sample compositions of time spent in sleep, sedentary behavior (SB), light physical activity (LPA) and moderate-to-vigorous physical activity (MVPA), at wave 0. Heat map depicts the frequency distribution of compositions.

**Additional file 4: Supplementary Table 1.** Compositional variation matrix. **Supplementary Table 2.** Cross-sectional associations between behaviors composition (proportion of the day spent in sleep, SB, LPA and MVPA) and mental health outcomes at wave 0 (sensitive analyses with prospective sample). **Supplementary Table 3.** Compositional isotemporal substitution analyses showing the hypothetical change on mental health indicators at wave 0 when 30-min of time is re-allocated between compositional behaviors (adjusted models 1 and 2). **Supplementary Table 4.** Compositional isotemporal substitution analyses projecting the theorical effect of reallocating 30-min of time between compositional behaviors on mental health indicators at wave 1 (adjusted models 1 and 2). **Supplementary Table 5.** Cross-sectional and prospective associations between behaviors composition (proportion of the day spent in sleep, SB, LPA and MVPA) and mental health outcomes (sensitive analyses excluding participants with middle or severe cognitive impairment -i.e., Mini-Mental State Examination score < 24).

## Data Availability

The datasets used and/or analyzed during the current study may be available from the Principal Investigator of the project (FR-A) upon reasonable request.

## Abbreviations

BMI: Body mass index
CoDA: Compositional Data Analysis
DAG: Directed Acyclic Graph
ENMO: Euclidean Norm Minus One
ES: Effect size
GDS-10: Geriatric Depression Scale – 10 items
ilr: Isometric log-ratio
kcal: Kilocalories
kg: Kilograms
LAW: Life After Work
LPA: Light physical activity
m: Meters
MCS: Mental component summary
MSSE: Mini-Mental State Examination
MVPA: Moderate-to-vigorous physical activity
NHANES: National Health and Nutrition Examination Survey
PA: Physical activity
QoL: Quality of life
SB: Sedentary behavior
SF-12: 12-Item Short Form Health Survey
STROBE: Strengthening the Reporting of Observational studies in Epidemiology
TV: Television
