# Supplementary figures and images for: Cross-sectional and prospective associations of sleep, sedentary and active behaviors with mental health in older people: a compositional data analysis from the Seniors-ENRICA-2 study
Source: Int J Behav Nutr Phys Act. 2021 Sep 16;18:124. doi: 10.1186/s12966-021-01194-9 (PMC8444566; doi:10.1186/s12966-021-01194-9)

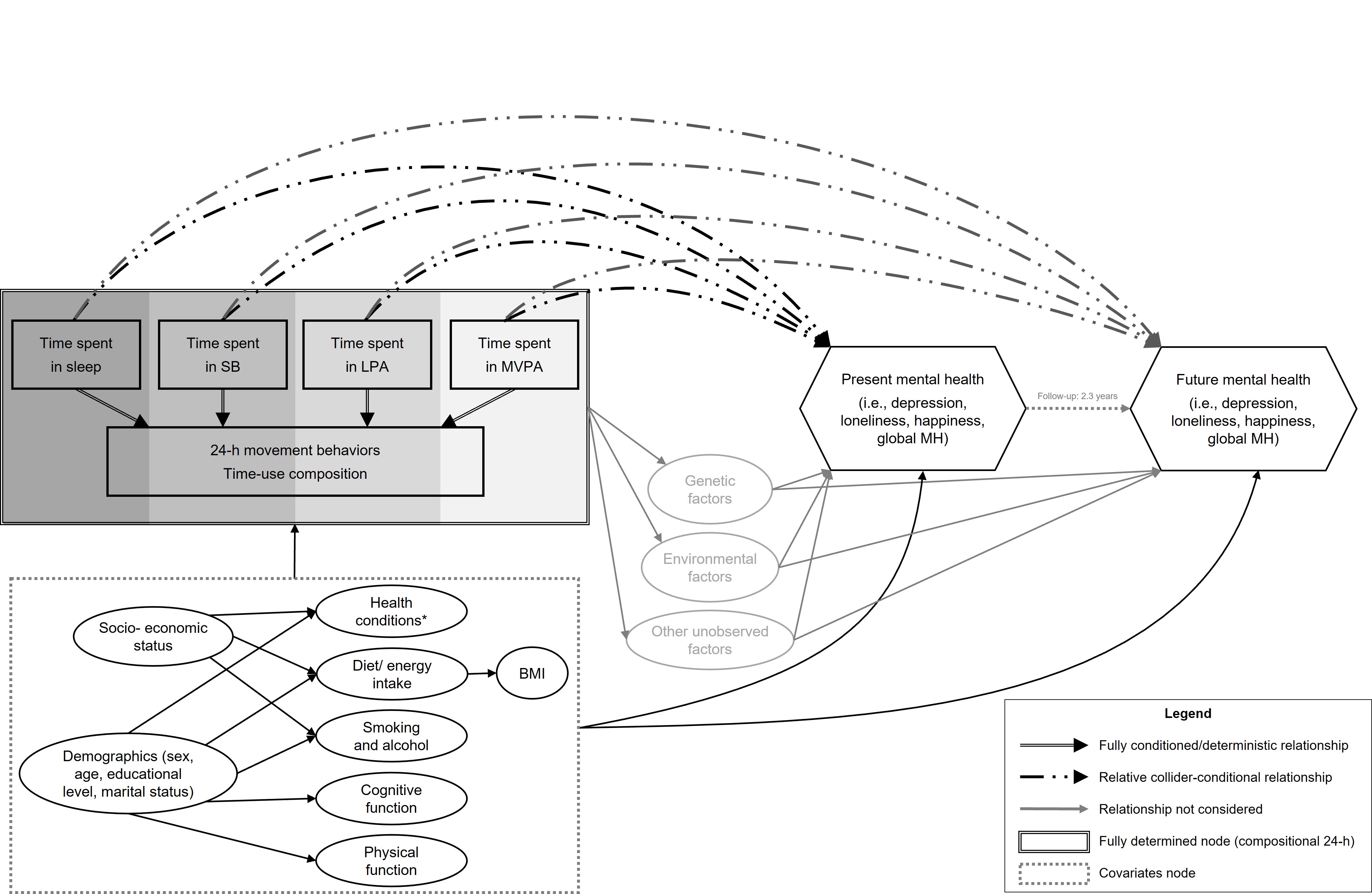

Supplement: Supplementary file 1 — Additional file 1: Supplementary Figure 1. Directed Acyclic Graph (DAG) illustrating the relational and causal assumptions of the present study. *Diagnosed health conditions included in our model were: cardiovascular disease, hypertension, diabetes mellitus, chronic respiratory disease, osteomuscular disease, neurodegenerative disease, and cancer at any site. The covariates node was created to show all confounding variables including in the model that have a potential relationship with both the 24-h composition and the present and future mental health indicators. Some relationships between factors were omitted for clarity. [file 12966_2021_1194_MOESM1_ESM.jpg]

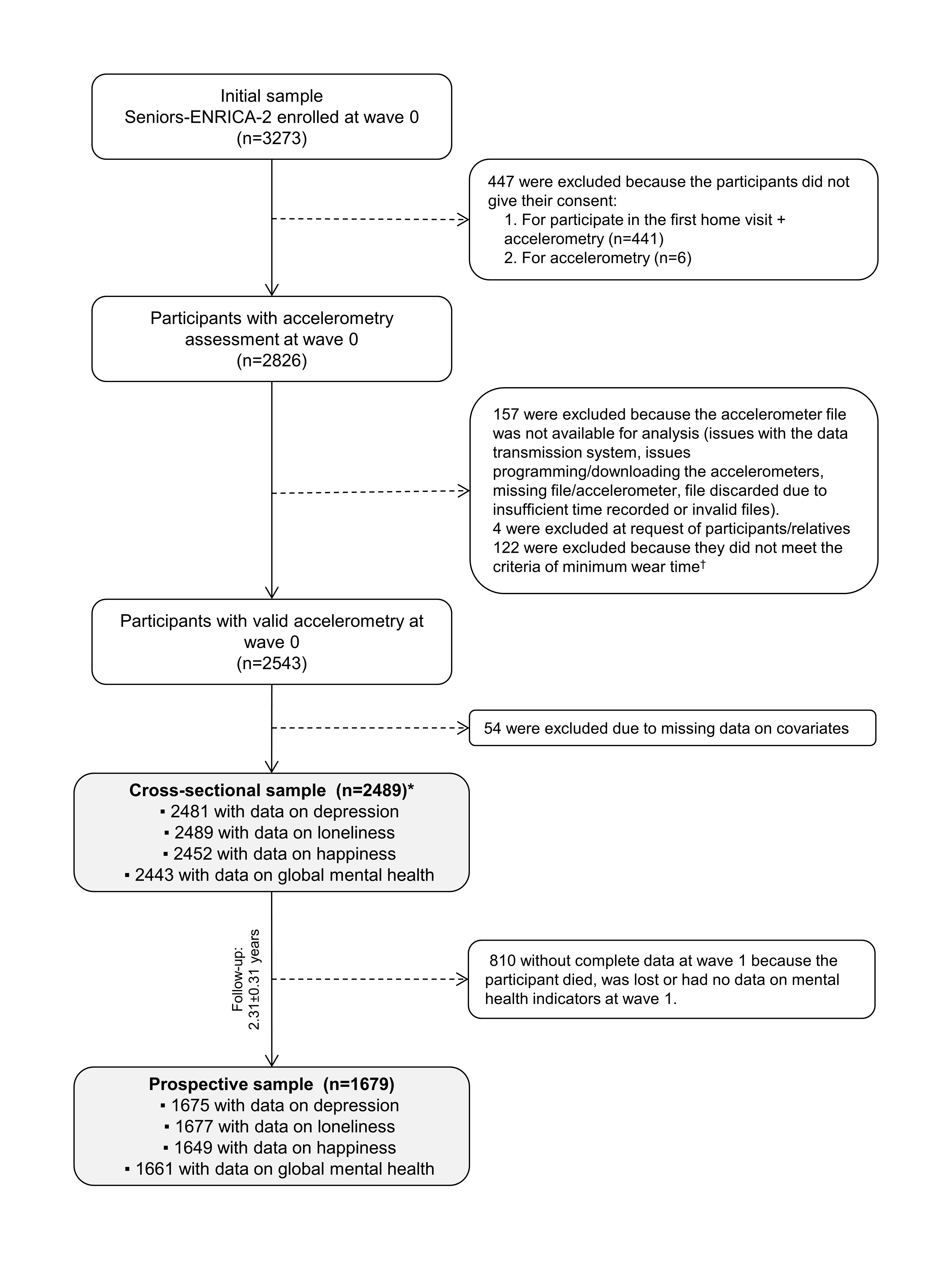

Supplement: Supplementary file 2 — Additional file 2: Supplementary Figure 2. Flowchart of the analytical cross-sectional and prospective sample for the present study, from the Seniors-ENRICA-2 participants. †The inclusion criteria were defined as having at least 4 valid days (at least, 1 valid weekend day; a valid day was considered as having at least 16 hours of record). *No significant differences were found between initial sample of Seniors-ENRICA-2 study and cross-sectional sample included in the present study in sex (46.87% vs. 46.93% of men, p = 0.965) or age (71.85±4.49 vs. 71.69±4.33, p = 0.161). [file 12966_2021_1194_MOESM2_ESM.jpg]

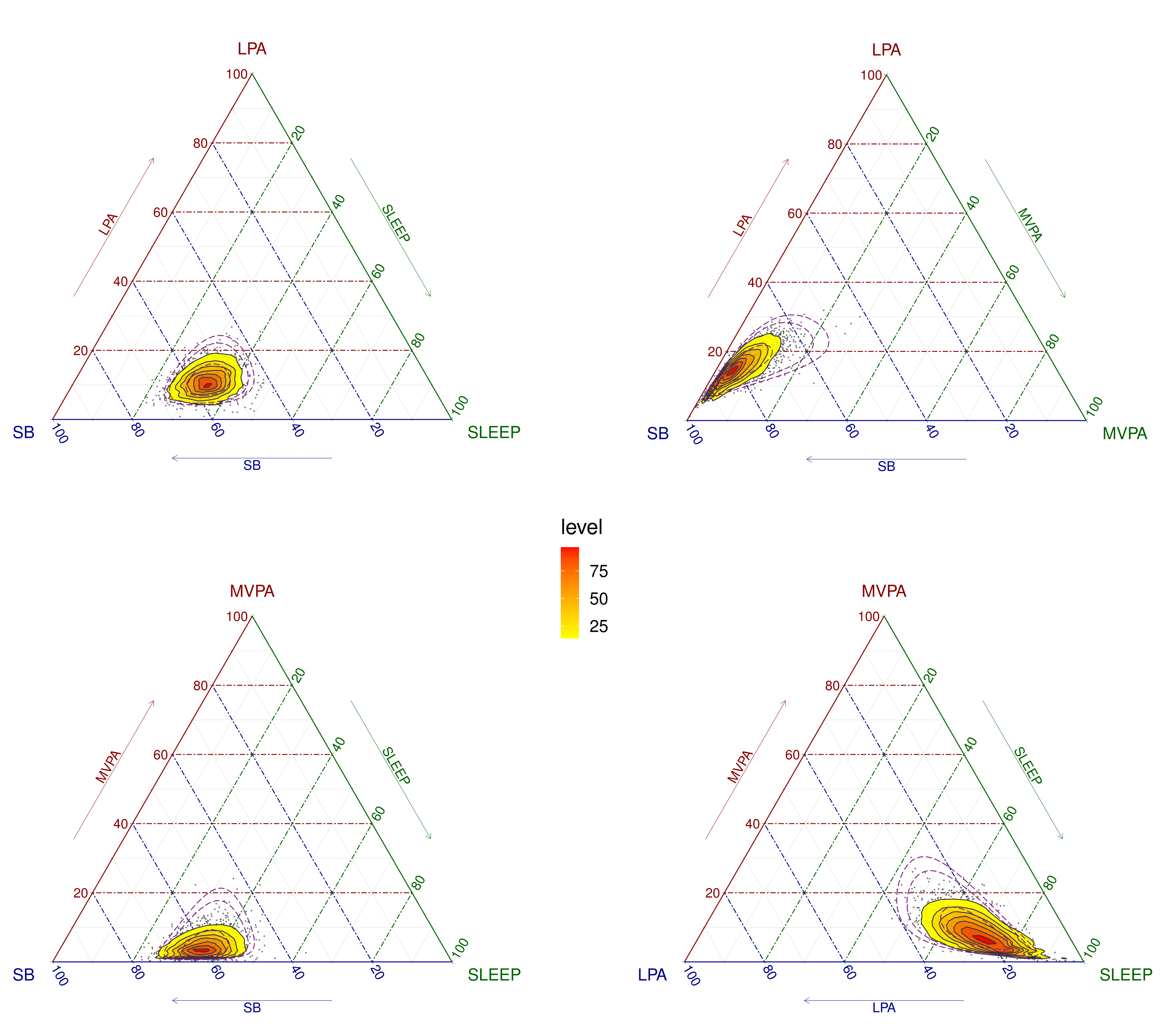

Supplement: Supplementary file 3 — Additional file 3: Supplementary Figure 3. Ternary plots of the prospective sample compositions of time spent in sleep, sedentary behavior (SB), light physical activity (LPA) and moderate-to-vigorous physical activity (MVPA), at wave 0. Heat map depicts the frequency distribution of compositions. [file 12966_2021_1194_MOESM3_ESM.png]
