# Supplementary material for: Cross-sectional and prospective associations of sleep, sedentary and active behaviors with mental health in older people: a compositional data analysis from the Seniors-ENRICA-2 study
Source: Int J Behav Nutr Phys Act. 2021 Sep 16;18:124. doi: 10.1186/s12966-021-01194-9 (PMC8444566; doi:10.1186/s12966-021-01194-9)
Supplement: Supplementary file 4 — Additional file 4: Supplementary Table 1. Compositional variation matrix. Supplementary Table 2. Cross-sectional associations between behaviors composition (proportion of the day spent in sleep, SB, LPA and MVPA) and mental health outcomes at wave 0 (sensitive analyses with prospective sample). Supplementary Table 3. Compositional isotemporal substitution analyses showing the hypothetical change on mental health indicators at wave 0 when 30-min of time is re-allocated between compositional behaviors (adjusted models 1 and 2). Supplementary Table 4. Compositional isotemporal substitution analyses projecting the theorical effect of reallocating 30-min of time between compositional behaviors on mental health indicators at wave 1 (adjusted models 1 and 2). Supplementary Table 5. Cross-sectional and prospective associations between behaviors composition (proportion of the day spent in sleep, SB, LPA and MVPA) and mental health outcomes (sensitive analyses excluding participants with middle or severe cognitive impairment -i.e., Mini-Mental State Examination score < 24). [file 12966_2021_1194_MOESM4_ESM.docx]

**Supplementary Table 1**. Compositional variation matrix

|  | Sleep | SB | Light PA | MVPA |
| --- | --- | --- | --- | --- |
| Cross-sectional sample (n=2489) | | | | |
| Sleep | 0 | 0.051 | 0.215 | 0.754 |
| SB | 0.051 | 0 | 0.228 | 0.806 |
| LPA | 0.215 | 0.228 | 0 | 0.362 |
| MVPA | 0.754 | 0.806 | 0.362 | 0 |
| Prospective sample (n=1679) | | | | |
| Sleep | 0 | 0.048 | 0.166 | 0.561 |
| SB | 0.048 | 0 | 0.183 | 0.618 |
| LPA | 0.166 | 0.183 | 0 | 0.308 |
| MVPA | 0.561 | 0.618 | 0.308 | 0 |

Values correspond to the log-ratio variance of each pair-wise behaviors. Values close to zero imply that two parts in the ratio are highly co-dependent. Abbreviations: SB: Sedentary Behavior; LPA: Light Physical Activity; MVPA: Moderate-to-Vigorous Physical Activity.

**Supplementary Table 2.** Cross-sectional associations between behaviors composition (proportion of the day spent in sleep, SB, LPA and MVPA) and mental health outcomes at wave 0 (sensitive analyses with prospective sample).

|  | Composition  model |  | Sleep | |  | SB | |  | LPA | |  | MVPA | |
| --- | --- | --- | --- | --- | --- | --- | --- | --- | --- | --- | --- | --- | --- |
|  | p |  | **γ** | p |  | **γ** | p |  | **γ** | p |  | **γ** | p |
| Depression (n=1676) |  |  |  |  |  |  |  |  |  |  |  |  |  |
| Model 1 | **<0.001** |  | 0.214 | *0.311* |  | -0.060 | 0.769 |  | 0.292 | *0.053* |  | -0.446 | **<0.001** |
| Model 2 | **<0.001** |  | 0.189 | 0.362 |  | -0.059 | 0.769 |  | 0.277 | *0.064* |  | -0.047 | **<0.001** |
| Model 3 | **<0.001** |  | 0.200 | 0.327 |  | -0.142 | 0.476 |  | 0.253 | *0.084* |  | -0.312 | **<0.001** |
| Loneliness (n=1679) |  |  |  |  |  |  |  |  |  |  |  |  |  |
| Model 1 | *0.052* |  | 0.137 | 0.454 |  | -0.154 | 0.384 |  | 0.208 | 0.111 |  | -0.190 | **0.007** |
| Model 2 | *0.065* |  | 0.082 | 0.635 |  | -0.139 | 0.412 |  | 0.234 | *0.060* |  | -0.178 | **0.008** |
| Model 3 | 0.145 |  | 0.063 | 0.714 |  | -0.118 | 0.480 |  | 0.207 | *0.092* |  | -0.152 | **0.021** |
| Happiness (n=1662) |  |  |  |  |  |  |  |  |  |  |  |  |  |
| Model 1 | **<0.001** |  | 0.014 | 0.953 |  | -0.239 | 0.289 |  | -0.063 | 0.706 |  | 0.288 | **0.001** |
| Model 2 | **0.002** |  | 0.017 | 0.939 |  | -0.210 | 0.345 |  | -0.058 | 0.725 |  | 0.251 | **0.004** |
| Model 3 | **0.006** |  | -0.006 | 0.980 |  | -0.195 | 0.378 |  | -0.013 | 0.936 |  | 0.214 | **0.014** |
| Mental health (n=1662) |  |  |  |  |  |  |  |  |  |  |  |  |  |
| Model 1 | 0.151 |  | -1.095 | 0.369 |  | 0.797 | 0.501 |  | -0.661 | 0.449 |  | 0.960 | **0.041** |
| Model 2 | 0.282 |  | -0.894 | 0.458 |  | 0.492 | 0.675 |  | -0.352 | 0.683 |  | 0.755 | 0.104 |
| Model 3 | 0.320 |  | -0.611 | 0.608 |  | 0.262 | 0.821 |  | -0.127 | 0.881 |  | 0.476 | 0.300 |

Values are shown as non-standardized gamma coefficients (γ) from CODA regression models; γ can be interpreted as the strength and direction of the association between the amount of time spent in one behavior relative to the others and the mental health outcome.

Abbreviations: SB: Sedentary Behavior; LPA: Light Physical Activity; MVPA: Moderate-to-Vigorous Physical Activity.

Model 1 adjusted by sex, and age; model 2 adjusted as model 1 plus educational level (no studies, primary, secondary, university), marital status (single, married, separated, widowed), and household economy (difficult economy, easy economy); model 3 adjusted as model 2 plus smoking status (current, former, never), alcohol consume (heavy, moderate, former, never), total energy intake (kcal/day), BMI (kg/m^2^), cognitive function (mini-mental state examination score), physical function (gait speed test score: unable, ≤0.43m/s, 0.44-0.60m/s, 0.61-0.77m/s, ≥0.78m/s), and chronic diseases (0, 1, 2+).

Statistically signiﬁcant values are shown in bold (p<0.05) and borderline signiﬁcant values are shown in italics (p<0.1).

**Supplementary Table 3.** Compositional isotemporal substitution analyses showing the hypothetical change on mental health indicators at wave 0 when 30-min of time is re-allocated between compositional behaviors (adjusted models 1 and 2).

|  |  |  | Depression  (n=2481) | |  | Loneliness  (n=2489) | |  | Happiness  (n=2452) | |  | Mental health  (n=2443) | |
| --- | --- | --- | --- | --- | --- | --- | --- | --- | --- | --- | --- | --- | --- |
|  |  |  | Model 1  ES (95% CI) | Model 2  ES (95% CI) |  | Model 1  ES (95% CI) | Model 2  ES (95% CI) |  | Model 1  ES (95% CI) | Model 2  ES (95% CI) |  | Model 1  ES (95% CI) | Model 2  ES (95% CI) |
| ↑ Sleep | ↓ SB |  | 0.018  (-0.012, 0.048) | 0.014  (-0.016, 0.044) |  | 0.005  (-0.019, 0.028) | 0.003  (-0.020, 0.026) |  | 0.002  (-0.030, 0.033) | 0.004  (-0.028, 0.035) |  | -0.087  (-0.261, 0.086) | -0.048  (-0.220, 0.125) |
|  | ↓ LPA |  | -0.049  (-0.113, 0.015) | -0.054  (-0.117, 0.009) |  | -0.033  (-0.083, 0.018) | -0.049  (-0.097, -0.001) |  | 0.017  (-0.049, 0.083) | 0.022  (-0.043, 0.087) |  | 0.009  (-0.355, 0.373) | -0.017  (-0.378, 0.343) |
|  | ↓ MVPA |  | **0.658**  **(0.52, 0.796)** | **0.613**  **(0.477, 0.748)** |  | **0.189**  **(0.079, 0.298)** | **0.184**  **(0.08, 0.289)** |  | **-0.384**  **(-0.527, -0.240)** | **-0.346**  **(-0.488, -0.204)** |  | **-1.386**  **(-2.153, -0.620)** | **-1.088**  **(-1.851, -0.325)** |
| ↑ SB | ↓ Sleep |  | -0.019  (-0.05, 0.012) | -0.015  (-0.046, 0.015) |  | -0.005  (-0.029, 0.020) | -0.003  (-0.026, 0.021) |  | -0.001  (-0.034, 0.031) | -0.003  (-0.035, 0.029) |  | 0.092  (-0.086, 0.270) | 0.051  (-0.126, 0.228) |
|  | ↓ LPA |  | **-0.067**  **(-0.131, -0.004)** | **-0.068**  **(-0.131, -0.005)** |  | -0.037  (-0.087, 0.013) | **-0.052**  **(-0.100, -0.004)** |  | 0.016  (-0.05, 0.082) | 0.018  (-0.047, 0.084) |  | 0.096  (-0.266, 0.458) | 0.031  (-0.328, 0.389) |
|  | ↓ MVPA |  | **0.640**  **(0.507, 0.772)** | **0.599**  **(0.468, 0.729)** |  | **0.184**  **(0.079, 0.290)** | **0.182**  **(0.081, 0.282)** |  | **-0.385**  **(-0.523, -0.248)** | **-0.349**  **(-0.486, -0.213)** |  | **-1.299**  **(-2.034, -0.565)** | **-1.040**  **(-1.771, -0.309)** |
| ↑ LPA | ↓ Sleep |  | 0.035  (-0.019, 0.089) | 0.04  (-0.013, 0.092) |  | 0.026  (-0.017, 0.068) | 0.040  (-0.001, 0.080) |  | -0.013  (-0.068, 0.043) | -0.017  (-0.072, 0.038) |  | 0.012  (-0.295, 0.320) | 0.027  (-0.277, 0.332) |
|  | ↓ SB |  | **0.054**  **(0.001, 0.107)** | **0.055**  **(0.002, 0.107)** |  | 0.030  (-0.012, 0.072) | **0.043**  **(0.003, 0.083)** |  | -0.011  (-0.066, 0.044) | -0.013  (-0.068, 0.041) |  | -0.080  (-0.381, 0.221) | -0.024  (-0.323, 0.275) |
|  | ↓ MVPA |  | **0.694**  **(0.519, 0.869)** | **0.653**  **(0.480, 0.826)** |  | **0.215**  **(0.076, 0.353)** | **0.224**  **(0.091, 0.357)** |  | **-0.397**  **(-0.578, -0.215)** | **-0.363**  **(-0.543, -0.183)** |  | **-1.379**  **(-2.355, -0.403)** | **-1.064**  **(-2.034, -0.094)** |
| ↑ MVPA | ↓ Sleep |  | **-0.335**  **(-0.405, -0.264)** | **-0.310**  **(-0.380, -0.241)** |  | **-0.095**  **(-0.151, -0.039)** | **-0.091**  **(-0.145, -0.037)** |  | **0.192**  **(0.118, 0.266)** | **0.172**  **(0.099, 0.245)** |  | **0.735**  **(0.334, 1.135)** | **0.572**  **(0.173, 0.97)** |
|  | ↓ SB |  | **-0.315**  **(-0.380, -0.251)** | **-0.295**  **(-0.359, -0.232)** |  | **-0.090**  **(-0.141, -0.039)** | **-0.088**  **(-0.137, -0.039)** |  | **0.194**  **(0.127, 0.260)** | **0.176**  **(0.109, 0.242)** |  | **0.642**  **(0.281, 1.004)** | **0.520**  **(0.161, 0.880)** |
|  | ↓ LPA |  | **-0.383**  **(-0.501, -0.264)** | **-0.363**  **(-0.48, -0.246)** |  | **-0.127**  **(-0.221, -0.033)** | **-0.140**  **(-0.23, -0.051)** |  | **0.209**  **(0.086, 0.332)** | **0.194**  **(0.072, 0.316)** |  | **0.739**  **(0.071, 1.406)** | 0.551  (-0.112, 1.214) |

Values are estimated modification on mental health indicators at wave 0 (effect size and 95% Confidence Interval) when 30 minutes were theoretically re-allocated from one behavior to another one, taking constant the time in the other behaviors.

Abbreviations: ES: Effect Size; SB: Sedentary Behavior; LPA: Light Physical Activity; MVPA: Moderate-to-Vigorous Physical Activity.

Model 1 adjusted by sex (male, female), age (years), and exposure outcome at baseline; model 2 adjusted as model 1 plus educational level (no studies, primary, secondary, university), marital status (single, married, separated, widowed), and household economy (difficult economy, easy economy).

Statistically signiﬁcant values are shown in bold.

**Supplementary Table 4.** Compositional isotemporal substitution analyses projecting the theorical effect of reallocating 30-min of time between compositional behaviors on mental health indicators at wave 1 (adjusted models 1 and 2).

|  |  |  | Depression  (n=1675) | |  | Loneliness  (n=1677) | |  | Happiness  (n=1649) | |  | Mental health  (n=1661) | |
| --- | --- | --- | --- | --- | --- | --- | --- | --- | --- | --- | --- | --- | --- |
|  |  |  | Model 1  ES (95% CI) | Model 2  ES (95% CI) |  | Model 1  ES (95% CI) | Model 2  ES (95% CI) |  | Model 1  ES (95% CI) | Model 2  ES (95% CI) |  | Model 1  ES (95% CI) | Model 2  ES (95% CI) |
| ↑ Sleep | ↓ SB |  | 0.027  (-0.003, 0.057) | 0.024  (-0.006, 0.055) |  | 0.005  (-0.021, 0.031) | 0.003  (-0.023, 0.029) |  | 0.005  (-0.029, 0.040) | 0.002  (-0.033, 0.037) |  | -0.057  (-0.258, 0.145) | -0.048  (-0.25, 0.154) |
|  | ↓ LPA |  | -0.010  (-0.072, 0.051) | -0.004  (-0.065, 0.057) |  | -0.031  (-0.083, 0.021) | -0.031  (-0.084, 0.021) |  | 0.025  (-0.046, 0.096) | 0.028  (-0.043, 0.099) |  | 0.247  (-0.163, 0.657) | 0.219  (-0.191, 0.629) |
|  | ↓ MVPA |  | **0.129**  **(0.008, 0.250)** | *0.116*  *(-0.005, 0.237)* |  | 0.096  (-0.007, 0.198) | 0.089  (-0.014, 0.192) |  | -0.097  (-0.235, 0.041) | -0.090  (-0.229, 0.049) |  | **-1.054**  **(-1.859, -0.249)** | **-1.024**  **(-1.831, -0.216)** |
| ↑ SB | ↓ Sleep |  | -0.028  (-0.059, 0.003) | -0.025  (-0.056, 0.006) |  | -0.005  (-0.032, 0.021) | -0.003  (-0.030, 0.024) |  | -0.005  (-0.041, 0.030) | -0.002  (-0.038, 0.034) |  | 0.058  (-0.149, 0.265) | 0.050  (-0.158, 0.257) |
|  | ↓ LPA |  | -0.037  (-0.098, 0.023) | -0.028  (-0.089, 0.032) |  | -0.036  (-0.088, 0.016) | -0.034  (-0.086, 0.018) |  | 0.020  (-0.05, 0.090) | 0.027  (-0.044, 0.097) |  | 0.303  (-0.102, 0.708) | 0.267  (-0.139, 0.673) |
|  | ↓ MVPA |  | 0.102  (-0.013, 0.217) | 0.092  (-0.023, 0.207) |  | 0.091  (-0.007, 0.188) | 0.086  (-0.012, 0.184) |  | -0.103  (-0.234, 0.029) | -0.092  (-0.224, 0.041) |  | **-0.998**  **(-1.766, -0.23)** | **-0.976**  **(-1.746, -0.206)** |
| ↑ LPA | ↓ Sleep |  | 0.004  (-0.048, 0.056) | -0.001  (-0.053, 0.052) |  | 0.025  (-0.020, 0.069) | 0.025  (-0.019, 0.070) |  | -0.021  (-0.081, 0.039) | -0.023  (-0.083, 0.037) |  | -0.193  (-0.542, 0.157) | -0.171  (-0.520, 0.178) |
|  | ↓ SB |  | 0.033  (-0.018, 0.083) | 0.025  (-0.026, 0.076) |  | 0.030  (-0.013, 0.073) | 0.028  (-0.015, 0.072) |  | -0.016  (-0.074, 0.043) | -0.022  (-0.080, 0.037) |  | -0.252  (-0.590, 0.087) | -0.221  (-0.561, 0.119) |
|  | ↓ MVPA |  | 0.134  (-0.020, 0.289) | 0.117  (-0.038, 0.271) |  | 0.121  (-0.011, 0.252) | 0.114  (-0.018, 0.246) |  | -0.118  (-0.295, 0.059) | -0.113  (-0.291, 0.065) |  | **-1.249**  **(-2.282, -0.217)** | **-1.196**  **(-2.231, -0.162)** |
| ↑ MVPA | ↓ Sleep |  | **-0.077**  **(-0.144, -0.010)** | **-0.070**  **(-0.137, -0.003)** |  | -0.052  (-0.109, 0.005) | -0.048  (-0.105, 0.010) |  | 0.050  (-0.027, 0.127) | 0.047  (-0.03, 0.124) |  | **0.575**  **(0.128, 1.022)** | **0.557**  **(0.109, 1.005)** |
|  | ↓ SB |  | -0.049  (-0.109, 0.011) | -0.044  (-0.105, 0.016) |  | -0.047  (-0.098, 0.005) | -0.045  (-0.096, 0.007) |  | 0.056  (-0.014, 0.125) | 0.049  (-0.021, 0.118) |  | **0.516**  **(0.114, 0.917)** | **0.507**  **(0.104, 0.909)** |
|  | ↓ LPA |  | -0.087  (-0.197, 0.024) | -0.073  (-0.183, 0.037) |  | -0.083  (-0.177, 0.011) | -0.079  (-0.173, 0.016) |  | 0.075  (-0.052, 0.202) | 0.075  (-0.052, 0.202) |  | **0.820**  **(0.082, 1.557)** | **0.774**  **(0.036, 1.513)** |

Values are estimated modification on mental health indicators at wave 1 (effect size and 95% Confidence Interval) when 30 minutes were theoretically re-allocated from one behavior to another one, taking constant the time in the other behaviors.

Abbreviations: ES: Effect Size; SB: Sedentary Behavior; LPA: Light Physical Activity; MVPA: Moderate-to-Vigorous Physical Activity.Abbreviations: SB: Sedentary Behavior; LPA: Light Physical Activity; MVPA: Moderate-to-Vigorous Physical Activity.

Model 1 adjusted by sex (male, female), age (years), and exposure outcome at baseline; model 2 adjusted as model 1 plus educational level (no studies, primary, secondary, university), marital status (single, married, separated, widowed), and household economy (difficult economy, easy economy).

Statistically signiﬁcant values are shown in bold (p>0.05).

**Supplementary Table 5.** Cross-sectional and prospective associations between behaviors composition (proportion of the day spent in sleep, SB, LPA and MVPA) and mental health outcomes (sensitive analyses excluding participants with middle or severe cognitive impairment -i.e., Mini-Mental State Examination score < 24).

|  | Composition  model |  | Sleep | |  | SB | |  | LPA | |  | MVPA | |
| --- | --- | --- | --- | --- | --- | --- | --- | --- | --- | --- | --- | --- | --- |
|  | p |  | **γ** | p |  | **γ** | p |  | **γ** | p |  | **γ** | p |
| Cross-sectional associations between behaviors composition and mental health outcomes at wave 0 | | | | | | | | | | | | | |
| Depression (n=2415) |  |  |  |  |  |  |  |  |  |  |  |  |  |
| Model 1 | **<0.001** |  | 0.322 | *0.078* |  | 0.062 | 0.728 |  | 0.191 | 0.152 |  | -0.576 | **<0.001** |
| Model 2 | **<0.001** |  | 0.262 | 0.142 |  | 0.067 | 0.706 |  | 0.199 | 0.127 |  | -0.528 | **<0.001** |
| Model 3 | **<0.001** |  | 0.259 | 0.142 |  | -0.029 | 0.868 |  | 0.176 | 0.172 |  | -0.406 | **<0.001** |
| Loneliness (n=2420) |  |  |  |  |  |  |  |  |  |  |  |  |  |
| Model 1 | **0.009** |  | 0.068 | 0.640 |  | -0.019 | 0.890 |  | 0.117 | 0.268 |  | -0.166 | **0.003** |
| Model 2 | **0.020** |  | 0.009 | 0.945 |  | -0.024 | 0.859 |  | 0.175 | *0.083* |  | -0.160 | **0.003** |
| Model 3 | 0.145 |  | -0.009 | 0.945 |  | -0.015 | 0.909 |  | 0.144 | 0.150 |  | -0.120 | **0.023** |
| Happiness (n=2387) |  |  |  |  |  |  |  |  |  |  |  |  |  |
| Model 1 | **<0.001** |  | -0.123 | 0.516 |  | -0.155 | 0.408 |  | -0.008 | 0.567 |  | 0.358 | **<0.001** |
| Model 2 | **<0.001** |  | -0.084 | 0.653 |  | -0.141 | 0.442 |  | -0.093 | 0.499 |  | 0.318 | **<0.001** |
| Model 3 | **<0.001** |  | -0.057 | 0.760 |  | -0.137 | 0.455 |  | -0.064 | 0.640 |  | 0.257 | **<0.001** |
| Mental health (n=2389) |  |  |  |  |  |  |  |  |  |  |  |  |  |
| Model 1 | **0.001** |  | -1.477 | 0.158 |  | 0.431 | 0.672 |  | -0.035 | 0.963 |  | 1.081 | **0.007** |
| Model 2 | **0.012** |  | -1.028 | 0.318 |  | 0.057 | 0.954 |  | 0.149 | 0.843 |  | 0.821 | **0.038** |
| Model 3 | 0.177 |  | -0.870 | 0.394 |  | 0.060 | 0.952 |  | 0.393 | 0.597 |  | 0.417 | 0.287 |
| Prospective associations between behaviors composition at wave 0 and mental health outcomes at wave 1 | | | | | | | | | | | | | |
| Depression (n=1650) |  |  |  |  |  |  |  |  |  |  |  |  |  |
| Model 1 | 0.189 |  | 0.313 | *0.082* |  | -0.270 | 0.123 |  | 0.056 | 0.532 |  | -0.100 | 0.153 |
| Model 2 | 0.252 |  | 0.280 | 0.117 |  | -0.219 | 0.209 |  | 0.027 | 0.833 |  | -0.088 | 0.197 |
| Model 3 | 0.244 |  | 0.341 | *0.054* |  | -0.311 | *0.055* |  | 0.026 | 0.834 |  | -0.057 | 0.405 |
| Loneliness (n=1652) |  |  |  |  |  |  |  |  |  |  |  |  |  |
| Model 1 | 0.324 |  | -0.021 | 0.890 |  | -0.001 | 0.996 |  | 0.130 | 0.239 |  | -0.108 | *0.068* |
| Model 2 | 0.352 |  | -0.049 | 0.750 |  | 0.026 | 0.860 |  | 0.124 | 0.257 |  | -0.101 | *0.084* |
| Model 3 | 0.360 |  | -0.049 | 0.747 |  | 0.027 | 0.854 |  | 0.122 | 0.264 |  | -0.100 | *0.088* |
| Happiness (n=1624) |  |  |  |  |  |  |  |  |  |  |  |  |  |
| Model 1 | 0.348 |  | 0.085 | 0.684 |  | -0.130 | 0.522 |  | -0.069 | 0.644 |  | 0.114 | 0.154 |
| Model 2 | 0.522 |  | 0.062 | 0.764 |  | -0.076 | 0.704 |  | -0.090 | 0.545 |  | 0.104 | 0.191 |
| Model 3 | 0.528 |  | -0.002 | 0.991 |  | -0.022 | 0.913 |  | -0.083 | 0.574 |  | 0.107 | 0.176 |
| Mental health (n=1638) |  |  |  |  |  |  |  |  |  |  |  |  |  |
| Model 1 | 0.148 |  | -1.050 | 0.383 |  | 0.931 | 0.427 |  | -0.877 | 0.309 |  | 0.995 | **0.032** |
| Model 2 | 0.157 |  | -0.982 | 0.412 |  | 0.784 | 0.501 |  | -0.764 | 0.373 |  | 0.962 | **0.037** |
| Model 3 | 0.290 |  | -0.753 | 0.527 |  | 0.693 | 0.550 |  | -0.778 | 0.361 |  | 0.838 | *0.067* |

Values are shown as non-standardized gamma coefficients (γ) from CODA regression models; γ can be interpreted as the strength and direction of the association between the change of time spent in one behavior relative to the others and the change in mental health outcome.

Abbreviations: SB: Sedentary Behavior; LPA: Light Physical Activity; MVPA: Moderate-to-Vigorous Physical Activity.

Model 1 adjusted by sex (male, female), age (years), and exposure outcome at baseline; model 2 adjusted as model 1 plus educational level (no studies, primary, secondary, university), marital status (single, married, separated, widowed), and household economy (difficult economy, easy economy); model 3 adjusted as model 2 plus smoking status (current, former, never), alcohol consume (heavy, moderate, former, never), total energy intake (kcal/day), BMI (kg/m^2^), cognitive function (mini-mental state examination score), physical function (gait speed test score: unable, ≤0.43m/s, 0.44-0.60m/s, 0.61-0.77m/s, ≥0.78m/s), and chronic diseases (0, 1, 2+).

Statistically signiﬁcant values are shown in bold (p<0.05) and borderline signiﬁcant values are shown in italics (p<0.1).
